# Association of Loneliness and Mindfulness in Substance Use Treatment Retention

**DOI:** 10.3390/ijerph20166571

**Published:** 2023-08-13

**Authors:** Johnathan M. Herczyk, Keith J. Zullig, Stephen M. Davis, Jennifer Mallow, Gerald R. Hobbs, Danielle M. Davidov, Laura R. Lander, Laurie Theeke

**Affiliations:** 1 Department of Social and Behavioral Sciences, School of Public Health, West Virginia University, Morgantown, WV 26506, USA; 2 Department of Health Policy, Management and Leadership, School of Public Health, West Virginia University, Morgantown, WV 26506, USA; 3 School of Nursing, West Virginia University, Morgantown, WV 26506, USA; 4 Department of Statistics, West Virginia University, Morgantown, WV 26506, USA; 5 Department of Behavioral Medicine and Psychiatry, Rockefeller Neurosciences Institute, School of Medicine, West Virginia University, Morgantown, WV 26506, USA; 6 School of Nursing, The George Washington University, Washington, DC 20052, USA

**Keywords:** loneliness, mindfulness, mental health, MOUD, retention, intervention strategies

## Abstract

Background: Elevated mental illness prevalence complicates efforts designed to address the opioid crisis in Appalachia. The recovery community acknowledges that loneliness impacts mood and engagement in care factors; however, the predictive relationship between loneliness and retention in medication-assisted outpatient treatment programs has not been explored. Our objectives were to identify associations between mental health factors and retention in treatment and elucidate treatment retention odds. Data were collected from eighty participants (*n* = 57 retained, *n* = 23 not retained) of a mindfulness-based relapse prevention (MBRP) intervention for individuals receiving medication for opioid use disorder (MOUD) in Appalachia. Loneliness, depression, and anxiety did not differ between the retained and not retained, nor did they predict not being retained; however, mindfulness was significantly lower among those not retained in treatment compared to those retained (OR = 0.956, 95% CI (0.912–1.00), and *p* < 0.05). Preliminary findings provide evidence for mindfulness training integration as part of effective treatment, with aims to further elucidate the effectiveness of mindfulness therapies on symptom reduction in co-occurring mental health disorders, loneliness, and MOUD treatment retention.

## 1. Introduction

The widespread distribution and misuse of opioids have resulted in the opioid crisis in the United States, which has been linked to nearly 1 million fatal overdoses since 1999 [1]. Despite consistent decreases in opioid prescribing patterns since 2012 [2] and the implementation of evidence-based strategies provided by the Centers for Disease Control and Prevention (CDC) to impede the continued escalation in overdose fatalities [3], increases in mortality persist. The U.S. recorded 105,800 fatal overdoses during the COVID-19 pandemic in 2021 [4], a 15% increase from the year prior [1].

Elevated rates of mental illness are complicating efforts to adequately address this dire public health issue. In 2021, 19.4 million U.S. adults had a co-occurring mental, behavioral, or emotional disorder with a substance use disorder [5]. A recent meta-analysis reports that 36.1% (95% CI 32.4–39.7%) of individuals with opioid use disorder (OUD) concurrently experienced depression, and 29.1% (95% CI 24.0–33.3%) experienced anxiety [6]. Loneliness, the perceived lack of social connections, is also significant to the experience of substance use and mental illness, owing to its influence on mood, motivation, and decision making [7].

### 1.1. Loneliness

Although frequently used interchangeably, social isolation and loneliness are two distinct, yet related, components of social connections. Social isolation is a deficiency in physical interactions between an individual and persons or systems in their social network [8,9]. On the other hand, loneliness is a discrepancy between the actual and perceived quality as well as availability of support from one’s relationships. Individuals may experience loneliness intermittently or chronically throughout their lives [8]; however, regardless of the experiential frequency, loneliness is detrimental to the health and survival of populations. The experience of loneliness is profound and prevalent throughout the United States, with a recent Surgeon General report highlighting the topic and providing a call to action. Loneliness and social isolation both impact and increase the risk of cardiovascular disease, stroke, dementia, and even premature death [10]. Evidence supports the idea that loneliness increases the risk of mood and anxiety disorders [10,11]. A systematic review into predictors of loneliness within SUD populations reports that people with substance use issues are lonelier than those without, and women as well as younger people with substance use issues may be lonelier than their peers [12]. A cross-sectional study on New England and Pacific Northwest opioid treatment programs found inverse relationships between men and women: Men with little to no loneliness were more likely to use illicit opioids than their severely lonely counterparts (OR = 2.86, 95% CI 1.15–7.14) [13]. In contrast, severely lonely women were more likely to use illicit opioids when compared to those with little to no loneliness (OR = 3.00, 95% CI 1.19–7.57) [13].

Mindfulness is a mental state of being fully present in the moment without judgment in addition to being aware of one’s surroundings and actions. This meditative practice has garnered credibility as a component of relapse prevention and treatment programs for substance use disorders because of the influence that mindfulness has on decision making, similar to loneliness. Mindfulness improves an individual’s awareness of internal and external stimuli that influence substance use behaviors; for populations with substance use disorders, this is focused on drug use triggers. Mindfulness-based relapse prevention (MBRP) interventions focus on improving individuals’ awareness to the current moment (e.g., drug use triggers) and adapting to recognize as well as control outcomes. Within populations with substance use disorders, MBRP has been shown to reduce depression, anxiety, and drug craving symptoms, as well as improve tolerance of challenging physical and emotional situations [14]. This supports the importance of considering mindfulness when assessing loneliness in substance-using populations. One such MBRP intervention within the Appalachian region has supported these notions [15] as well as identified an inverse correlation between mindfulness and loneliness among individuals diagnosed with OUD [16].

### 1.2. Mental Health in Central Appalachia

Appalachia is a region of the Eastern United States, marked by the Appalachian Mountains, which stretch from southern New York to northern Mississippi. Although Appalachia is comprised of 13 states in total, West Virginia is uniquely situated in Central Appalachia and is the only state fully within the region. Overall, the Central Appalachian region suffers from poor social determinants of health (e.g., income, education, and employment), and this is amplified for the 35.5% of the population living in rural areas of the state [17].

Overall mental health quality is lower among Central Appalachians. Extensive research has found loneliness to be pervasive and detrimental to quality of life among middle-aged [18] and older, chronically ill, Appalachians [19]. Moderate loneliness is hypothesized to be widespread yet under-reported by adults living in the region due to the Appalachian cultural value of self-reliance [20] as well as the isolating geography of the region [21]. Physical barriers and rurality limit access to care and opportunities for social connections [21], thus contributing to the prevalence of loneliness in Central Appalachia. Furthermore, rates of depression are 17% higher [22]. Surveillance data continue to report that West Virginia has the highest age-adjusted opioid overdose mortality rate of 90.9 per 100,000 people in 2021, an increase of nearly 40 points compared to prepandemic rates [23]; however, the region remains underserved by mental health providers and designated as a health professional shortage area [24], with additional access barriers being introduced by state legislation, such as prohibitions on new methadone treatment programs [25].

The recovery community has long noted the influence that loneliness has on mood, motivation [7], and decision making, as well as engagement in managing one’s health [11]. A recent intervention has shown promise for reducing loneliness by employing mindfulness techniques [26], a concept that has also shown promise for reducing drug craving, depression, and anxiety among individuals receiving medication for opioid use disorder (MOUD) [15]. Although loneliness has been associated with an increased likelihood of using illicit opioids [13] and, thus, subsequently experiencing a period of relapse, the predictive relationship between loneliness and retention in MOUD outpatient treatment has not been explored, particularly in Appalachian populations.

Because the initial services are difficult to access, retaining individuals in MOUD treatment is critical to improve outcomes within the region. Retention in treatment is essential to avoiding relapse, overdose, and death, with longer retention improving mortality rates [27,28,29,30]. The prevalence of retention in treatment is considerably variable in the recent literature, ranging from 19% to 91% over twelve months [27,28]. Consistent definitions of retention are uncommon [31], making it difficult to compare findings across interventions; therefore, no best practice has been reported to increase retention in MOUD [28,31], despite multiple attempts to address known barriers to retention [32,33,34]. No study has been found to have investigated the influence of loneliness on retention in treatment within Appalachia.

Therefore, the current study’s objectives were as follows: (1) identify associations between mental health factors and demographics with retention in MOUD treatment, and (2) elucidate the odds of retention in MOUD treatment due to perceived loneliness and other mental health diagnoses. It was hypothesized that higher perceptions of loneliness would decrease the odds of retention.

## 2. Materials and Methods

This secondary analysis utilized data obtained from participants at the start of their participation in an MBRP intervention at an outpatient comprehensive opioid addiction treatment (COAT) program at a large university located in the Central Appalachian region. Biweekly medication management and group therapy are included in the COAT program model, as is weekly attendance at community-based self-help meetings (e.g., Narcotics Anonymous). The COAT program combines cognitive behavioral therapy with MOUD, as appropriate. Medication management relies on the use of Suboxone^®^, or buprenorphine, which is a safe and effective partial agonist prescribed by physicians to control drug cravings and withdrawal symptoms, with a lower potential for misuse or abuse by an individual with OUD [35]. Individuals were recruited from the COAT program during the intermediate stage (at least 90 consecutive days of sobriety) and required to be 18 years or older, have received a diagnosis of opioid use disorder over the past year (using DSM-5 criteria), obtained a 12-step sponsor, and comprehend as well as communicate in English. Conversely, exclusion criteria for the MBRP intervention included risk of suicide, risk of incarceration, and/or having a psychotic disorder or comorbid diagnosis that prevented engagement in a mindfulness intervention. All of the exclusion criteria were assessed by the treating physician through the COAT program.

At the time of recruitment, individuals were provided the option of receiving MBRP plus MOUD or continuing cognitive behavioral therapy treatment as usual (TAU) plus MOUD for a 24-week intervention conducted between September 2017 and December 2019. MBRP is a noninvasive and nonpharmacologic 8-week treatment that targets an array of factors (e.g., awareness of triggers and cravings, mindfulness in high-risk situations and daily life, acceptance, self-care, etc.) For a complete description of the recruitment procedures and the MBRP intervention, see Zullig et al. [15].

### 2.1. Measures and Data Collection

All of the individuals that contributed self-reported demographic and mental health data at the time of enrollment (baseline) were included in this investigation. Mental health data were also collected after 12 weeks, 24 weeks, and 36 weeks postintervention using the following measures but were unused in the current study. Loneliness was assessed using the 20-item Revised UCLA Loneliness Scale (R-UCLA), which consists of Likert scale questions (11 negative and 9 positive statements that are reverse-coded). The R-UCLA has established validity, with Cronbach’s alpha estimates ranging from 0.89 to 0.94, and an average test–retest reliability estimate (*r* = 0.73) [36]. Participants respond to each item to describe their feelings; the available responses are 1 = Never, 2 = Rarely, 3 = Sometimes, or 4 = Often. The range of possible scores is 20–80; higher scores indicate greater loneliness. Sample statements include “I am unhappy doing so many things alone” and “I have nobody to talk to.” The baseline internal consistency estimate for the R-UCLA in this study was 0.92.

Depression was assessed using the 5-item Overall Depression Severity and Impairment Scale (ODSIS), and anxiety was assessed with the 5-item Overall Anxiety Severity and Impairment Scale (OASIS). For the ODSIS, summing each item score produces a total score, with potential scores ranging from 0 to 20; scores greater than or equal to 8 are used to determine a diagnosis of depression. The ODSIS demonstrates good estimates of convergent and discriminant validity across clinical and nonclinical samples [37]. The OASIS is scored similarly, with the sum of each item and total potential score ranging from 0 to 20. On the OASIS, scores ≥ 8 are also used to determine a diagnosis of anxiety [37]. The scale demonstrates excellent reliability and convergent as well as divergent validity (α = 0.80) in both clinical and nonclinical samples [38]. The baseline internal consistency estimates for the ODSIS and OASIS in this study were 0.89 and 0.92, respectively.

Mindfulness was operationalized using the Five-Facet Mindfulness Questionnaire (FFMQ). The FFMQ contains 39 items across five subscales (observing, describing, acting with awareness, accepting without judgment, and nonreactivity). It has demonstrated evidence of good internal consistency, with alpha values from 0.76 to 0.91 [39]. The response options for each item are 1 = Never or very rarely true, 2 = Rarely true, 3 = Sometimes true, 4 = Often true, or 5 = Very often or always true. The FFMQ is scored by summing each subscale score into a total score; values for the total score range from 39 to 195, with higher scores indicating greater mindfulness. The baseline internal consistency estimate for the FFMQ total scale in this study was 0.89 [39].

Retention in treatment was defined as remaining in either TAU or MBRP throughout the 36-week data collection period. As part of COAT clinic standards at the time of this study, individuals who missed two consecutive sessions were considered not retained and subsequently dismissed. Demographic variables included age at baseline, gender (male/female), marital status (single, married, and divorced or separating), education (non-high school, high school or GED, and any college), employment (full-time, part-time, and unemployed), and insurance coverage (Medicaid, Medicare, private insurance, and no insurance). *Race* was dichotomized as either white or other to mirror the results of the most recent United States census for West Virginia, where 93.5% of the state’s population was Non-Hispanic White [40]. The study from which deidentified data were obtained for secondary analysis was approved by the Institutional Review Board of West Virginia University.

### 2.2. Analysis

Data were analyzed using JMP^®^ 16 (SAS Institute Inc., Cary, NC, USA). Descriptive statistics were calculated for all of the demographic and mental health variables. Fisher’s exact tests were used to explore associations between demographic variables and retention in treatment. Pearson and point biserial correlation analyses were used to identify correlations between retention in treatment and each mental health variable. Two sample *t*-tests were employed to identify differences in mean age, and mental health scores between those retained and not retained were investigated with Student’s *t*-tests. The significance level for all of the analyses was set at α ≤ 0.05.

Multiple logistic regression was used to elucidate the odds of dropping out or being dismissed from treatment and an individual’s loneliness after adjustment for potential confounders at any point during the 36-week data collection period. The small sample size constrained the number of predictors that could be reliably considered in the final model, with the least frequent event, termination of treatment, observed 22 times [41]. Relaxing the 10 events per predictor norm to 5–9 events per predictor has been supported for use in logistic regression; therefore, 4 predictors were included in the model [42]. The inclusion of predictors was driven by the hypothesis, with loneliness automatically being included. The inclusion of depression and anxiety [7,19], as well as mindfulness [15], was informed by the currently available literature as well as the identified associations between the psychometric variables measured.

## 3. Results

### 3.1. Sample Demographics

A total sample of 89 individuals contributed data at the baseline; however, nine participants were excluded from the current analyses due to not meeting the inclusion criteria set forth for the larger MBRP intervention. Therefore, eighty individuals were included in the current analysis, where 57.5% (*n* = 46) identified as female. The mean age of the participant population was 36 years (SD: 8.9 years), and most of the participants identified as white (*n* = 75, 94%). Over half (57%) of the participants were either employed full-time (*n* = 31) or part-time (*n* = 15). Bivariate analyses (Table 1) showed that there was a statistically significant association between retention and gender (*p* < 0.001), education (*p* < 0.001), employment status (*p* < 0.001), insurance provider (*p* < 0.001), and race (*p* < 0.001). Despite these associations, none of these variables were significantly predictive of retention in MOUD when logistically modeled and, thus, were not considered for inclusion.

Moderate to severe loneliness was highly prevalent (R-UCLA ≥ 40) within the study population at the baseline, with a mean score of 49 (SD: 5.1, range of 36 to 64). Fifty-one of the participants (89%) that were retained in treatment were classified as lonely, and 21 (95%) of those that were not retained in MOUD were lonely. The mean depression score within the population was 5.9 (SD: 4.4), with 33 (41%) of the total participants having an ODSIS score greater than or equal to eight, indicative of depression. Severe anxiety (OASIS ≥ 10) was prevalent in 32 (40%) of the enrolled population. Bivariate analyses (Table 2) showed no differences (*p* > 0.05) in mean loneliness, depression, nor anxiety between those that were retained and those that discontinued treatment; however, mean mindfulness was significantly higher (*p =* 0.018) among those retained in treatment (mean: 125, SD: 12) than among individuals that discontinued treatment (mean: 117, SD: 14).

### 3.2. Correlations between Mental Health Variables and Retention

Pearson correlations were computed to identify relationships among the mental health predictor variables measured at the baseline. Statistically significant positive correlations between all of the predictors (*p* < 0.05) were observed, with the largest positive correlation observed between anxiety and depression, r (65) = 0.72, *p* < 0.001. Point biserial correlations revealed no statistical correlation between retention in MOUD and loneliness (r_pb_ = 0.059, *p* = 0.599), anxiety (r_pb_ = −0.027, *p* = 0.811), nor depression (r_pb_ = 0.022, *p* = 0.843); however, these calculations did reveal a statistically significant, yet small, positive association between retention in MOUD and mindfulness (r_pb_ = 0.28, *p* = 0.012).

### 3.3. Logistic Regression

The predictor variable loneliness was found to not statistically contribute to the model (β = 0.0237, SE = 0.045, L-R χ^2^ = 0.27, and *p* = 0.601). Likewise, anxiety (β = 0.0209, SE = 0.052, L-R χ^2^ = 0.16, and *p* = 0.685), depression (β = −0.0245, SE = 0.052, L-R χ^2^ = 0.22, and *p* = 0.637), and mindfulness (β = 0.0237, SE = 0.045, L-R χ^2^ = 0.27, and *p* = 0.601) were not individually correlated with retention in treatment. Results from the adjusted model are reported in Table 3. A lack of fit analysis (χ^2^ = 73 (62), *p* = 0.153) suggests that the model fit the data well. The results of the regression analysis revealed that, while holding other predictors constant, the odds of not being retained in MOUD were not statistically influenced by loneliness (OR = 0.926, 95% CI (0.792–1.08), and *p* = 0.333), depression (OR = 1.01, 95% CI (0.836–1.22), *p* = 0.921), or anxiety (OR = 1.07, 95% CI (0.887–1.29), *p* = 0.476).

However, there was a marginally significant difference in the odds of not being retained in MOUD between individuals who reported greater perceived mindfulness compared to individuals who reported reduced perceived mindfulness. Specifically, individuals who reported greater perceived mindfulness (as measured by the FFMQ scores) were 4% less likely to discontinue MOUD than their counterparts who reported reduced perceived mindfulness (OR = 0.956, 95% CI (0.912–1.00), and *p* < 0.05) after accounting for other mental health constructs.

## 4. Discussion

The current analysis investigating the capabilities of loneliness perceived by individuals prior to participating in an MBRP intervention and the intermediate stage of treatment to predict the cessation of treatment did not yield statistically significant results; however, promising findings indicate that greater mindfulness increased retention in MOUD treatment and may present a treatment option to be incorporated into standard-of-care pharmacological interventions.

Perceived loneliness did not differ between those that were retained in MOUD compared to those that were not. Likewise, when accounting for depression and anxiety, two co-occurring mental health issues shown to be correlated with loneliness [19], the odds of treatment cessation were not statistically significant. Therefore, loneliness was not found to be a predictor of retention in the current model. Despite the nonsignificant findings related to loneliness, additional, longitudinal, and randomized investigations should incorporate and re-evaluate the influence of this perception on retention in treatment. Loneliness is recognized to be directly related to substance abuse [12,13] and drug craving [16]. It is important to distinguish between loneliness and social support or isolation; inconsistent definitions of loneliness have conflated these two concepts [12], making it more difficult to detect the true influence of this perception of the availability and quality of relationships. Regardless of the null results of loneliness to predict retention in treatment, loneliness was clinically prevalent in this population and aligns with the increased national prevalence observed over the same period.

A national survey conducted in July 2019 found the prevalence of loneliness to be 61% among Americans aged ≥ 18 years [43], a 7% increase from the previous year [44]. This was exacerbated postpandemic. Further surveillance conducted in 2021 reported young adults feeling lonely and left out 2.5 times (46%, 18 years–34 years) more than older adults (16%, ≥ 55 years) [45]. These recent estimations not only exemplify that loneliness is prevalent [43], but also that it is increasing postpandemic [44]. Recognizing the prevalence of loneliness and the numerous influences that it has on physical and chronic disease [7,8,19,46], mental health [10,11,18,20], and substance misuse [12,13] supports the need for further investigations into interventions addressing loneliness. Additional investigations should further focus on loneliness among Appalachians, particularly those receiving MOUD, with the aim of developing interventions for incorporation into OUD treatment programs.

Statistically, mindfulness marginally demonstrated the capacity to predict retention in treatment in our sample. In the current study, more mindful individuals were 4% less likely to not be retained in MOUD treatment. This observation may be due to participants’ resiliency, or the ability to manage challenging thoughts, feelings, situations, and overall adversity. Resiliency plays a critical protective role for individuals during the treatment and recovery process; however, it is highly influenced by a person’s own experiences. Mindfulness has been shown to promote resiliency and reduce maladaptive coping within college-aged students [47]. Becoming more mindful indicates one’s awareness and ability to focus on internal as well as external triggers [14]; this may subsequently increase one’s ability to adapt and enhance inherent resiliency, ultimately leading to retention in treatment. Although resiliency was not measured and considered in the current work, the relationship between it and mindfulness among individuals receiving MOUD should be further explored and considered, with a focus on effective mindfulness-based interventions.

Even though there is support for joint MOUD, counseling, and behavioral therapies [48], there remains no recommendation for the most effective therapy combination for individuals with OUD. Zullig et al. [15] supports the notion that mindfulness-based therapy can be effectively integrated into MOUD and successfully reduce drug craving, depression, and anxiety. This aligns with previous work conducted on a second model of mindfulness-based therapy, mindfulness-oriented recovery enhancement (MORE) [49]. Additionally, MBRP has shown potential to reduce loneliness among individuals in the intermediate stage of MOUD [16]. The current study provides additional evidence to also suggest that MBRP may improve OUD treatment retention. Retaining individuals in MOUD treatment programs is vital to reduce relapse and, subsequently, prevent overdoses as well as death [27]. Preliminary findings from the current study suggest that mindfulness may serve as a predictive indicator of discontinuing treatment.

Uniform definitions of retention are uncommon, making it difficult to compare findings across interventions; therefore, no best practice has been reported to increase retention in MOUD [28,31]. Integrating mindfulness-based therapies and actively monitoring this practice may prove effective at improving retention and reducing overdoses as well as mortality in OUD populations. This notion is supported by the American Society of Addiction Medicine, whose updated practice guidance encourages the integration of psychosocial and environmental assessments in conjunction with pharmacotherapies in treatment programs [50]. The current results provide preliminary evidence for mindfulness training integration as a component of effective treatment; however, since there is still not evidence to support the most effective behavioral therapy, it is recommended that future endeavors elucidate the effectiveness of MBRP versus MORE therapies with regard to reductions in co-occurring mental health disorder symptoms, loneliness, and influence on retention in treatment.

Consistent with a recent meta-analysis [51], no gender differences were observed between the retention groups, although significant (*p <* 0.001) associations were noted between retention in MOUD and gender, education, employment, insurance, marital status, and race; however, these social determinants of health and demographics were not statistically significant in predicting individuals not being retained in treatment. Future studies may wish to further explore, with an adequate sample size, the impact of social determinants of health, loneliness, and mindfulness on retention in MOUD.

### Limitations

Findings from the current study are subject to certain limitations. A small sample size in the total enrolled population ultimately limited the number of individuals discontinuing treatment, thus reducing the potential generalizability. This also narrowed the ability to truly understand the impact of loneliness on retention and treatment, which bears the need for further investigation. Although predictors in the logistic regression model were selected based on theoretical considerations from previous work and clinical relevance, high correlations between some predictors suggest collinearity, which may have diluted the effect of each predictor on the outcome. Additionally, although only a single adjusted regression model was tested, conducting a Bonferroni correction for multiple comparisons would show marginally significant results for mindfulness; however, this does not negate the clinical importance of mindfulness and the role it plays during the treatment and recovery process; thus, further investigation is warranted. Additionally, participants in the MBRP intervention were not randomized into groups, and the intervention itself was quasi-experimental. Hence, selection bias cannot be discredited. Finally, nearly 94% of the participants identified as white, therefore limiting its generalizability to ethnically diverse populations and regions; however, this is representative of the Appalachian region.

## 5. Conclusions

Recognizing the prevalence of loneliness and the health outcomes associated with its perception [19,46] is an important consideration in the treatment of Appalachian adults receiving MOUD treatment. Assessments of loneliness in the current study were conducted pre-COVID-19. Estimates of loneliness in the general population increased 2.5 times in 2021 compared to 2019 [43,45]; therefore, it is assumed that perceptions of loneliness also worsened within MOUD populations. Furthermore, although loneliness was not significantly predictive of retention in treatment, moderate loneliness was still observed within this population of patients in the intermediate stage of outpatient MOUD treatment, thus supporting a further need to investigate this discrepancy between the actual and perceived quality as well as availability of support from one’s relationships.

Moreover, acknowledging that mindfulness may offer a broad array of benefits, including increasing retention in MOUD, reducing co-occurring mental health issues [15,16,49], improving resiliency [47], and decreasing loneliness [16] further provides evidence of the potential benefits that mindfulness-based therapies offer adults in MOUD. Incorporating mindfulness-based therapies into treatment programs may address the prevalence of loneliness, reduce relapsing as well as overdosing, and ultimately save lives. Further investigation to explore barriers to access and retention in treatment experienced by individuals with OUD is warranted.

## Figures and Tables

**Table 1:** Demographic associations with, differences between, and predictability of retention.

Variable	Total (*n* = 80)	Retained (*n* = 57)	Not Retained (*n* = 22)	Bivariate *p*-Value	Regression *p*-Value
^‡^ Age				0.836	0.859
Mean (SD)	36 (8.9)	36 (10)	35 (5.4)		
Range	23–67	23–67	23–45		
^†^ Gender				*<0.001*	0.159
Male	37	27 (47%)	10 (45%)		
Female	41	29 (50%)	12 (55%)		
Other	2	2 (3%)			
^†^ Education				*<0.001*	0.559
Non-high school	6	5 (9%)	1 (5%)		
High school or GED	47	35 (61%)	12 (54%)		
Any college	26	17 (30%)	9 (41%)		
^†^ Employment				*<0.001*	0.416
Full-time	31	21 (36%)	10 (45%)		
Part-time	15	10 (17%)	5 (23%)		
Not employed	34	27 (47%)	7 (32%)		
^†^ Insurance				*<0.001*	0.140
Medicaid	64	45 (79%)	19 (86%)		
Medicare	5	5 (9%)	-		
Other	10	7 (12%)	3 (14%)		
^†^ Marital Status				*<0.001*	0.601
Single	46	33 (57%)	13 (59%)		
Married	16	11 (19%)	5 (23%)		
Divorced or separating	18	14 (24%)	4 (18%)		
^†^ Race				*<0.001*	0.120
White	75	54 (96%)	21 (95%)		
Other	3	2 (4%)	1 (5%)		

^†^ Fisher’s exact test; ^‡^ two-tailed *t*-test. Italic is to denote statistical significance.

**Table 2:** Mean (SD) scores and bivariate results of mental health variables.

Mental Health Variable	Total	Retained (*n* = 57)	Not Retained (*n* = 22)	*p*-Value
Anxiety (OASIS)				
^‡^ Mean (SD) score	7.7 (4.4)	7.6 (4.2)	7.9 (5.1)	0.812
Range	0–19	0–15	0–19	
Depression (ODSIS)				
^‡^ Mean (SD) score	5.9 (4.4)	5.9 (4.1)	5.7 (5.2)	0.843
Range	0–18	0–16	0–18	
Loneliness (R-UCLA)				
^†^ Mean (SD) score	49 (5.1)	49 (5.4)	48 (4.5)	0.609
Range	36–64	36–64	38–56	
* Mindfulness (FFMQ)				
^‡^ Mean (SD) score	123 (13)	125 (12)	117 (14)	*0.018*
Range	78–147	86–147	78–146	

^†^ One-tailed *t*-test; ^‡^ two-tailed *t*-test; and * *p* < 0.05.

**Table 3:** Adjusted logistic regression results.

Predictor	β	Std Error	L-R χ^2^	*p*-Value	OR	95% CI
Anxiety	0.0681	0.095	0.51	0.476	1.07	0.887–1.29
Depression	0.0096	0.096	0.01	0.921	1.01	0.836–1.22
Loneliness	−0.0773	0.079	0.94	0.333	0.926	0.792–1.08
* Mindfulness	−0.0451	0.024	3.6	0.047	0.956	0.912–1.00

* *p* < 0.05.

## Data Availability

The datasets used and/or analyzed during the current study are available from the corresponding author upon reasonable request.
